# Ultra-High Efficiency and Broad Band Operation of Infrared Metasurface Anomalous Reflector based on Graphene Plasmonics

**DOI:** 10.1038/s41598-018-37562-y

**Published:** 2019-02-04

**Authors:** Sina Soleymani, M. Zeki Güngördü, Patrick Kung, Seongsin M. Kim

**Affiliations:** Electrical and Computer Engineering Department, University of Alabama, Tuscaloosa, 35487 USA

## Abstract

Infrared metasurface anomalous reflector with ultra-high efficiency and broad band operation is designed via multi-sheet graphene layer with triangular holes. The anomalous reflection angle covers the range of 10° to 90° with the efficiency higher than 80%, over a broad spectral range from 7 μm–40 μm of infrared spectrum. It reaches above 92% at the center wavelength in the spectral response. By increasing the periodicity of phase gradient, we can expand this frequency band even further without losing efficiency. The compact design of metasurface affords the adjustability of the electrochemical potential level of graphene by means of gating. Additionally, the impact of the number of graphene sheets for the optimum efficiency of the proposed structure is investigated. By adding the secondary graphene metasurface with opposite direction of phase gradient, we demonstrated the tunability of the reflection angle from *θ*_*r*_ to −*θ*_*r*_ with bias voltage.

## Introduction

Due to their exotic manner of manipulation of electromagnetic waves, metasurfaces have recently become an essential research topic in the optics and photonics fields^1–4^. Anomalous refraction and reflection of light with these extraordinary surfaces has been utilized in several newly developed devices such as polarimetry, flat lenses, holography, surface plasmon couplers, solar sails, etc^5–8^, as the prominent applications of metasurfaces^9–13^. As controlling the light propagation in an embedded structure becomes a vital aim, one significant breakthrough for many viable application is to actively manipulate the direction of light, i.e. reflection or refraction by means of external voltage. The rapid progress of improvement and innovative design has become possible for various metasurfaces with the optically exotic graphene^14–20^. Graphene and other 2D materials might be one of the best candidates for designing externally controllable metasurfaces. By changing their electrochemical potential level, it is possible to change the optical characteristics (i.e. the surface conductivity) in an extraordinary fashion^21,22^.

Plasmonic metasurfaces with a phase gradient have the ability to alter and tilt the wavefront of electromagnetic waves^23–26^. In this regard, the structures with low quality factors are the center of interests^27–30^. Broadband operation and higher efficiency of anomalous reflection are significant factors in realizing flexible metamirrors^31,32^. In parallel, trilayer metal-insulator-metal (MIM) metasurfaces with noble metals provide the efficient reflection in the desired direction^27,30,33^. Recently, triangularly shaped plasmonic metallic metasurfaces demonstrated continuous phase gradient configuration^34,35^. In an alternative design, magnetically polarized particles have shown an excellent plasmonic metasurface-like behavior with P-polarized electromagnetic waves^36^.

In numerous studies, however, metasurfaces that operate at fixed frequencies or in extremely narrow bands suffer low efficiencies in the other frequency bands^27^. Besides, cross-polarized metasurfaces do not offer high efficiencies useful for many applications like the solar sails^4^. Due to the overlapping of Fabry-Perot and the antenna resonances in the spectra of reflection, some metasurfaces act like an absorber metamaterial rather than a mirror, which results in a decrease of the anomalous reflection efficiency^20,37–39^. Efficiency of metamirrors can be easily defined by the power ratio of an anomalously reflected (i.e. reflected in predicted and desired direction) electromagnetic wave to the incident wave. The frequency band of operation is mainly determined by the generalized Snell’s law and the optical properties of materials. An additional influential factor contributing to the anomalous reflection is the near-field interaction of metasurface with the incident electromagnetic wave.

In this paper, infrared multi-layered graphene metasurfaces controllable with bias voltages are designed to achieve exceptional high efficiencies^40,41^. The structure studied here is sensitive to linearly polarized electromagnetic waves. Each graphene ribbon has a specific width, chosen to have a certain plasmon resonance frequency. This provides the strong near-field interaction, and therefore, highly efficient anomalous reflections. Both the periodicity of the phase gradient direction (x-direction) and the periodicity of the polarization direction (y-direction) are considered to design a structure and to investigate the behavior of its reflection accordingly with the assistance of the Finite-difference time-domain (FDTD) method. It is worth mentioning that similar structure with the trapezoid shaped silver plasmonic antennas array operating in the visble light spectrum has been reported by other research group^42^. However, unlike silver plasmon reflector, the reflection efficiency of our graphene based reflector can have more than 80% for wide wavelength band. Moreover, by leveraging the tunability of the Fermi level of the graphene sheets^43–47^, we propose uniquely tunable graphen plasmonic reflector structure in this work.

## Results and Discussions

### Design of Graphene Plasmonic Metasurface

**Figure 1:**
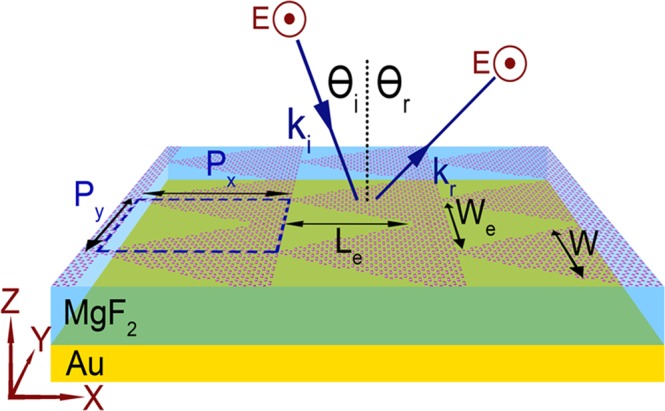
Metasurface based on multi-sheet graphene layer with triangular holes. The unit cell with the periodicities of P_x_ and P_y_ is encircled with the blue dashed line. The phase gradient is in the x-direction and sensitive to TE polarization. The dielectric material of cavity is MgF_2_ with the thickness of 2.5 μm. W and W_e_ are the width of triangular graphene ribbons and width of the triangular eatches, respectively. The gold reflector is considered at the other side of the cavity reflecting almost 100% of electromagnetic wave. The considered dimensions for one of the first case is ***P***_***x***_ = **20.1** ***μm*** and ***P***_***y***_ = **2.2** ***μm***, with an etched triangle base of ***W***_***e***_ = **2** ***μm*** and a length of ***L***_***e***_ = **20** ***μm***.

The proposed metasurface reflector consists of a multi-sheet graphene layer on top of the dielectric material, followed by the Au ground plane as illustrated in Fig. 1. MgF_2_ with the refractive index of 1.38 is a dielectric layer sandwiched between a graphene multilayer and a metal ground plane. The imaginary part of the refractive index of the MgF_2_ is negligible in the discussed wavelength band^18,48^. The main objective of the dielectric material in our theoretical analysis is to provide a cavity for the metasurface, and the considered material could be replaced with a material with a similar refractive index. The thickness of the dielectric layer is 2.5 μm.

Triangular holes etched through the graphene layer produce the phase gradients required for anomalous reflection. The dimensions of a unit-cell of the metasurfaces reflector are *P*_*x*_ = 20.1 *μm* and *P*_*y*_ = 2.2 *μm*, with an etched triangle base of *W*_*e*_ = 2 *μm* and a length of *L*_*e*_ = 20 *μm*. It is worthy to mention that size of the unit cell is set slightly larger (about 0.1–0.2 um) than the etched triangular hole, to make graphene layers be connected. This design is proposed since the etching of graphene can be more practical than producing triangular graphene islands in the fabrication process. However, the designs based on triangular islands, with specific electrochemical potential level could yield the similar results. In this paper the dimensions of the unit cell and etching dimensions are chosen in order to produce ultra-high efficiency metasurfaces and to make it practical one might need to manipulate the proposed dimensions, due to the fabrication process restrictions.

It is expected the width, *W*, and the periodicity, *P*_*y*_ of the triangular shape of graphenes (TSGs) in our model has a significant impact on tuning the supported plasmon resonance frequencies. It also affects its Lorentz like phase changes^34^. Since the width, *W* of TSG increases in x-direction, the resonance frequencies shift to lower frequencies through the phase gradient.

The plasmon resonance frequency of graphene nano-ribbons (GNRs) with fixed *W* is given by^38,39^,

1 $${\omega }_{0}=\frac{e}{{\rm{\hslash }}}\sqrt{\frac{NP\mu }{{\varepsilon }_{0}({\varepsilon }_{a}+{\varepsilon }_{s})W}}$$

where, *N*, *P*, and *μ* represent the number of graphene sheets in each ribbon, the width and periodicity related factor, and the electrochemical potential level, respectively.

This relation could be easily derived from the circuit model of graphene ribbons^37,38^. In the denominator, the *W* represents the width of GNRs, which applied to TSG in our model, varies in the y-direction. The effective permittivity of the graphene environment can be calculated from the permittivity of Air (ε_*a*_) and substrate (ε_*s*_). We can consider the average value of *P* is equal to 0.65 for our TSGs, since *P* = *qW*/*π*, where q is the first eigenvalue produced from the mode solution of GNRs^37^. An additional effective factor of the resonance and phase gradient is *μ*, where it could be tuned by gating or doping. By increasing the carrier density of the graphene layer, we can blue-shift the resonance frequency. This provides an important degree of freedom in adjusting the phase gradient to a specific frequency band as well as enhancing the reflection efficiency for a specific spectrum. The value of *μ* is considered to be 250 meV for each graphene sheet. Changing the value of chemical potential does not have an effect in changing the reflection angle. However, by manipulation of *μ*, we can intensify or diminish the anomalous reflection efficiency and even obscure this behavior. The number of graphene sheets, *N*, included in Eq. (1), is based on the fact that only an inductive impedance of graphene is proportionally correlated to *N*^38,39^.

Eq. (2) expresses the generalized Snell’s law of reflection^27^,

2 $$\sin ({\theta }_{r})-\,\sin ({\theta }_{i})=\frac{\nabla {\varphi }_{x}}{{k}_{0}}$$

Since ∇*ϕ*_*x*_ = 2*π*/*P*_*x*_, this indicates that only the length of the phase gradient unit (*P*_*x*_) and the free space wavelength of light are important in determining the angle of reflection. On the other hand, the periodicity in the y-direction (*P*_*y*_) and *W* determine the operating band where the phase gradient starts and ends.

**Figure 2:**
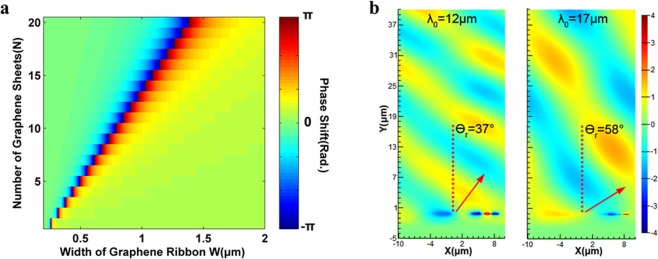
(**a**) Phase shift over the metasurface with the value of ***W*** increasing in the x-direction, considering the impact of number of graphene sheets. The free space wavelength considered to be ***λ***_0_ = **10** ***μm*** here. (**b**) Reflection of the plane wave from the metasurface with the wavelengths of ***λ***_0_ = **12** ***μm*** and ***λ***_0_ = **17** ***μm***. The direction of incident electromagnetic wave propagation is normal to the metasurface (***θ***_***i***_ = **0**°). The unit-cell of this metasurface is ***P***_***x***_ = **20.1** ***μm***, and ***P***_***y***_ = **2.2** ***μm***.

The Fig. 2(a) demonstrated the reflected electromagnetic wave covers a 2π phase range due to a continuous phase shift over one unit cell of TSG metasurface along the *P*_*x*_. As *W* increases, the wavelength of resonance increases proportionally. Furthermore, increasing the Fermi energy level of the graphene layer blue-shifts the resonance wavelength. Therefore, as the number of graphene sheets increases, the Lorentz like phase change shifts to the longer *W* side. In the case of having only one graphene layer as shown in the Fig. 2(a), a variation of the phases along the *P*_*x*_ is restricted to shorter *W* side, so that the phase shift over the unit-cell becomes almost zero, ∇*ϕ*_*x*_ ≈ 0. In addition, increasing *N* helps to provide a stronger near-field interaction of light. By increasing the number of the graphene sheets (*N*) the number of the conducting channels and the total number of the carriers will be increased, which intensifies the plasmonic response of the material, and the plasmon field intensity (i.e. the plasmonic near-field intensity)^40^. Additionally, it is experimentally proved that multilayer graphene structures provide broader tunability of the Fermi level, comparing to the single layer graphene structure^41^. In this regard, we purposely chose 20 graphene layers. We examined our proposed structure with different simulation methodologies (1) scaling the conductivity of a single layer graphene up to 20, compared with (2) considering 20 graphene sheets with a spacing of 5 nm to verify the consistency of our results. Figure 2(a) represents the impact of *N* on the Lorentz like phase shift along the metasurface.

From the expression of $\sin \,{\theta }_{r}-\,\sin \,{\theta }_{i}={\lambda }_{0}/{P}_{x}$, we can estimate that by increasing the free space wavelength of the electromagnetic wave, the reflection angle will increase until it reaches,*λ*_0_ = *P*_*x*_, where we can observe the conversion of propagating waves to surface waves. Figure 2(b) compares the wavefronts of the reflected plane wave for the free space wavelengths of *λ*_0_ = 12 *μm* and *λ*_0_ = 17 *μm* with reflecting angles of *θ*_*r*_ = 37°, and *θ*_*r*_ = 58°, respectively. It shows that the reflection angle increases with wavelength, while the efficiency stays almost the same. The anomalous reflection efficiency for these two cases stays over 85%, which will be discussed in the next section.

### Analysis of Efficiency and Reflection Properties

**Figure 3:**
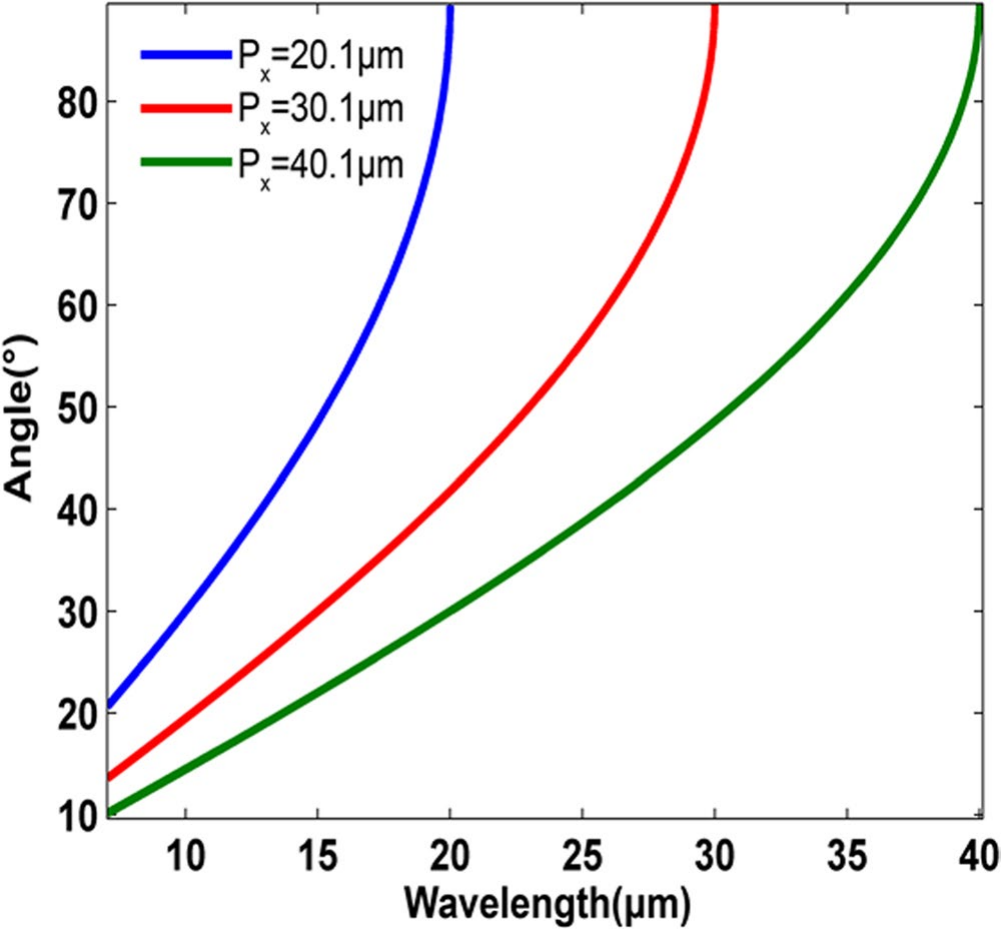
Anomoulous reflection angle for various phase gradient periodicities of ***P***_***x***_ = **20.1** ***μm***, ***P***_***x***_ = **30.1** ***μm***, and ***P***_***x***_ = **40.1** ***μm***. The periodicity in y-direction is ***P***_***y***_ = **2.2** ***μm***. In addition, the incident electromagnetic wave angle is ***θ***_***i***_ = **0**°.

Next, we examined the effect of the periodicity of *P*_*x*_, and *P*_*y*_ on the overall operating wavelength bandwidth of the TSG metasurface reflector. The cut-off wavelength of anomalous reflection can be analytically predicted by both the surface conductivity of graphene and the generalized Snell’s law, simultaneously. According to Eq. (2) this metasurface is operable up to the maximum wavelength equal to *P*_*x*_, the periodicity of phase gradient unit cell. This relation is depicted in Fig. 3 for different *P*_*x*_ values.

The minimum wavelengths limit (*λ*_*s*_) is determined by the intra-band term of the imaginary part of the conductivity function (${\sigma }_{i}^{intra}$) of graphene, which gradually drops at shorter wavelengths, and ablates near field interaction. The ${\sigma }_{i}^{intra}$ represents the metallic behavior of the graphene in infrared and Terahertz bands. The cut-off wavelength of the surface plasmons of the graphene is below the zero-crossing wavelength of ${\sigma }_{i}^{intra}$. Changing the Fermi energy level of graphene can shift the surface plasmon wavelength to either the longer or shorter wavelengths. In this case, surface plasmon wavelength of the graphene sheets with a specific Fermi energy level is responsible for the shorter cut-off wavelength of the proposed metasurfaces. The value of the electrochemical potential level of the graphene sheets is assumed to be 250 meV.

**Figure 4:**
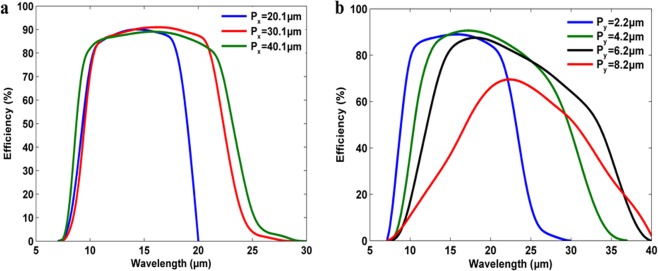
(**a**) Anomolous reflection efficiency for the phase gradient periodicities of ***P***_***x***_ = **20.1** ***μm***, ***P***_***x***_ = **30.1** ***μm*** and ***P***_***x***_ = **40.1** ***μm***, with blue, red and green colors. The periodicity in y-direction is ***P***_***y***_ = **2.2** ***μm***.The incident electromagnetic wave propagation direction is normal to the metasurface (***θ***_***i***_ = **0**°). (**b**) Anomolous reflection efficiency for the phase gradient periodicity of ***P***_***x***_ = **40.1** ***μm***. The periodicities in y-direction are ***P***_***y***_ = **2.2** ***μm***, ***P***_***y***_ = **4.2** ***μm***, ***P***_***y***_ = **6.2** ***μm***, and ***P***_***y***_ = **8.2** ***μm*** with blue, green, black and red colors, respectively. The incident electromagnetic wave propagation direction is normal to the metasurface (***θ***_***i***_ = **0**°). The value of reflection angle ***θ***_***r***_ increases with wavelength as it is demonstrated in Fig. 3.

Figure 4(a) depicts the efficiency of anomalous reflection over the wide wavelength band for various periodicity lengths, *P*_*x*_. The reflection angle covers the broad wavelength band of 7 μm to 20 μm for unit-cell dimensions of *P*_*x*_ = 20.1 *μm* and *P*_*y*_ = 2.2 *μm*. According to Eq. (2), as the periodicity increases in the x-direction (i.e. phase gradient direction) the supported maximum cut-off wavelength (*λ*_*L*_) should red-shift. For instance, the cut-off wavelength for the above unit-cell dimensions is *λ*_*L*_ = 20.1 *μm*, and this device is practically operable up to the predicted cut-off wavelength. Additionally, the metasurface with unit-cell of *P*_*x*_ = 30.1 *μm* and *P*_*y*_ = 2.2 *μm* is predicted to have the *λ*_*L*_ = 30.1 μm, which effectively supports up to *λ*_*L*_ = 27 *μm*. The metasurface with the periodicities of *P*_*x*_ = 40.1 *μm* and *P*_*y*_ = 2.2 *μm* supports effectively up to *λ*_*L*_ = 29 *μm*, while the predicted maximum cut-off wavelength is 40.1 μm.

As it is shown in the Fig. 4(a), the efficiency of metasurface reflector increases sharply to 80% at a wavelength of 10 μm, and stays with an efficiency over 80% up to the wavelength of 18 μm. The efficiency reaches as high as 92% at *λ* = 14 *μm*. In other words, this unit-cell can reflect with the angles of 30° to 65° with an efficiency of higher than 80%. In the same regard, the metasurface with longer phase gradient periodicity *P*_*x*_ = 30.1 *μm* supports the wavelength band from 7 μm to 27 μm, while its efficiency stays higher than 80% from 10 μm to 21 μm. The efficiency of this metasurface exceeds 90% at the middle of the operational band. Accordingly, the metasurface with the *P*_*x*_ = 40.1 *μm*, supports the widest wavelength band from 7 μm up to 29 μm, with an efficiency higher than 80% over the wide band of 10 μm to 22 μm. These results exhibit high efficient anomalously reflecting metasurface reflector with flexible choice of wavelengths by choosing the proper physical dimensions of the phase gradient periodicity (P_x_).

The second physical dimension, which could be manipulated in order to achieve wider operational wavelength band is *P*_*y*_, which is periodicity in y-direction is equal to 2.2 μm. Since the value of *W* in Eq. (1) determines the resonance wavelength of the metasurface, by increasing it, the resonance wavelength supported by the unit-cell red-shifts. By increasing the *P*_*y*_ and *W*, simultaneously, the maximum cut-off wavelength (*λ*_*L*_) is red-shifted. However, this value could not exceed the predicted cut-off wavelength of Snell’s law (i.e. Eq. (2)) for specific *P*_*x*_.

Figure 4(b) shows the results of the efficiencies of anomalous reflection over the wide wavelength band for various periodicity lengths, *P*_*y*_, with fixed value of *P*_*x*_ = 40.1 *μm*. With the *P*_*y*_ = 2.2 *μm*, the operating wavelength band is from *λ*_*s*_ = 7 *μm* to *λ*_*L*_ = 29 *μm*. However, with increasing values of *P*_*y*_ = 4.2 *μm* and *P*_*y*_ = 6.2 *μm*, the maximum cut-off wavelength are red-shifted to *λ*_*L*_ = 36 *μm*, and *λ*_*L*_ = 39 *μm*, respectively. Further, the maximum cut-off wavelength of *λ*_*L*_ = 40.1 *μm* is achieved with *P*_*y*_ = 8.2 *μm*, which is expected from Eq. (2).

Increasing the periodicity in y-direction tends to support the anomalous reflection over the wider band of operation, but the efficiency of the metasurface decreases in general. For instance, the metasurface with the unit-cell dimensions of *P*_*y*_ = 2.2 *μm* and *P*_*x*_ = 40.1 *μm* supports the wavelength band from 10 μm to 22 μm with the efficiency of more than 80%. In case of *P*_*y*_ = 4.2 *μm*, and *P*_*y*_ = 6.2 *μm*, both support the efficiencies of 80% in the wavelength range from 12 μm to 24 μm, and from 15 μm to 24 μm, respectively. However, with *P*_*y*_ = 8.2 *μm*, the maximum efficiency reaches only 70% at 24 μm.

As it is discussed extensively, the reduction of the efficiency in the longer wavelengths is due to the approaching to the plasmonic cut-off wavelength or the cut-off wavelength supported by the Snell’s law. However, there is a general reduction of the efficiency in the longer wavelength, which is due to the cavity thickness (as same as in the case with *P*_*y*_ = 6.2 *μm*, and *P*_*x*_ = 40.1 *μm*). Our simulation results disclosed that by increasing the thickness of the cavity we can reach the higher efficiency in the longer wavelengths at the same time of losing the efficiency in the shorter wavelengths.

It is clear that increasing the bandwidth of operation (by means of increasing the unit-cell dimensions) results in wider spanning range of the reflection angle. For instance, the total spatial range supported by the metasurface with periodicities of *P*_*x*_ = 20.1 *μm* and *P*_*y*_ = 2.2 *μm* is 20° to 90°; however, this range is expanded to 10° to 90° when the periodicities are *P*_*x*_ = 40.1 *μm* and *P*_*y*_ = 8.2 *μm*, in spite of the reduction in the efficiency.

### Tunable Metasurface Anomalous Reflector

**Figure 5:**
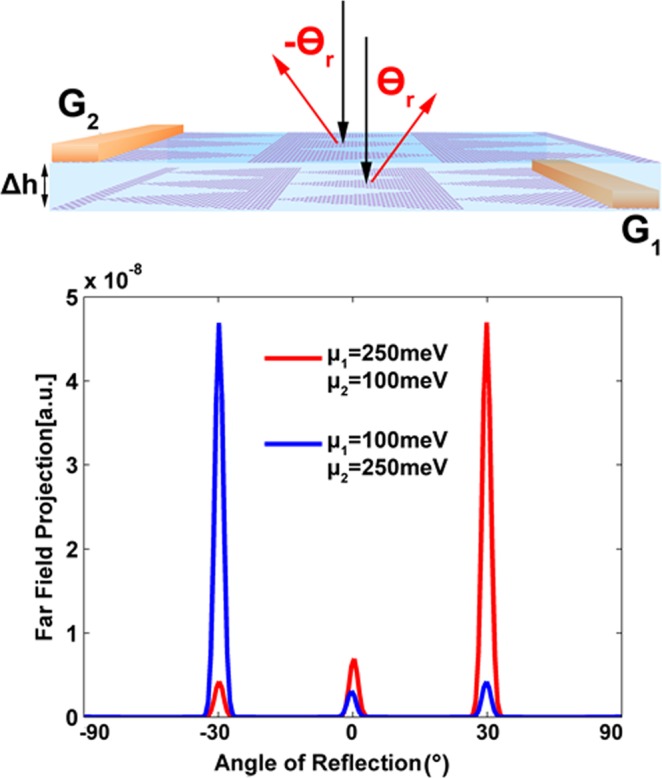
Placing double graphene layers with the oposite pahse gradient directions and producing a metamirror with controlabe reflection in ***θ***_***r***_ and −***θ***_***r***_directions. The incident electromagnetic wave propagation is normal to the metasurface (***θ***_***i***_ = **0**°) and the free space wavelength is ***λ***_0_ = **10** ***μm***. The unit-cell dimensions of this metasurface is ***P***_***x***_ = **20.1** ***μm*** and ***P***_***y***_ = **2.2** ***μm***.

As it is feasible to manipulate the anomalous reflection by means of changing the *μ* of graphene sheets, we add a second reflecting metasurface with phase gradient in the opposite direction. Figure 5 presents the tunable anomalous reflection by shifting the electrochemical potential by gating bias voltage. The two reflecting metasurface must be close to each other with small length of separation (Δ*h*) comparing to the thickness of cavity. Each graphene metasurface is formed from 20 sheets of graphene layer and these two metasurfaces are supported by the same cavity and ground metal plane as shown in the Fig. 5. In view of the metasurface configuration demonstrated in Fig. 1, and replacing the single phase gradient layer with two opposite phase gradients layers, we can implement this tunable device. In this simulation, the value of Δ*h* is considered to be 50 nm, which is very small comparing to the cavity thickness of 2.5 μm. By implementing the gates inside of the dielectric media or outside of the metasurface or changing the doping level of the graphene sheets, one can change the Fermi level of the graphene phase gradients. By reducing the *μ*_1_ to 100 meV, the first phase gradient metasurface loses its functionality and becomes transparent. But due to the second phase gradient metasurface, with *μ*_2_ = 250 meV and opposite direction of phase gradients, the reflection angle changes to −*θ*_*r*_. This kind of functionality would bring flexibility in controlling the direction of reflectors at any frequency.

However, adding another phase gradient metasurface reduces the efficiency near to 50%. This reduction is due to the strong absorption of the transparent graphene layer with *μ* = 100 *meV* and also amplified absorption of two metasurfaces with 20 graphene sheets^21^. In spite of this absorption, 50% efficiency of anomalous reflection still provides better performance than several other metasurface configurations which do not have tunability with external voltage^4^.

The results also reveal that the polarization of the electromagnetic wave must be perpendicular to the direction of the phase gradient. One can use several phase gradients in different directions in order to have a design supporting anomalous reflection for different polarization directions of the TE waves. This device could be actively controlled with the method illustrated in Fig. 5. Exciting TM surface plasmonic electromagnetic waves of the graphene is also possible, however, in this case the incident wave couples to the surface wave in the resonance frequency, which is different from the case of the TE polarized wave.

## Conclusion

We designed an anomalously reflecting infrared metasurface with exceptional high efficiency and a wide frequency band of operation via triangularly etched multi-sheet graphene layers. Our design achieved the anomalous reflection angle, covering a range of 10° to 90° with an efficiency higher than 80% over a broad spectral range from 7 μm–40 μm. The efficiency of the anomalous reflection exceeds 92% at the center wavelength. In this design, the effective phase gradient is generated from the triangular shape on the graphene layers. This phase gradient and its gradual changes determined the anomalous reflection angles and reflection efficiency in the range of infrared incident wavelength. The maximum cut-off wavelength (*λ*_*L*_) of the metasurface is determined by the phase gradient periodicity (*P*_*x*_). In contrast, the minimum cut-off wavelength (*λ*_*S*_) is due to the surface conductivity and the surface plasma frequency of the graphene layer. Graphene is an excellent candidate for a tunable metasurface reflector, as the surface conductivity of graphene can be controlled by means of external gating voltage. We found that adjustability of the Fermi level of graphene was not able to change the anomalous reflection direction from a phase gradient metasurface, but can possibly either disable or activate the anomalous reflection operation. Finally, we demonstrated tunability of the reflection angle from *θ*_*r*_ to −*θ*_*r*_ by separately controlling the electrochemical potential levels of double phase gradient layers embedded in opposite directions. In this case, one of the phase gradients is disabled by reducing the Fermi level to 100 meV, and the other layer with reverse phase gradient direction is activated by increasing its Fermi level to 250 meV. This novel configuration of the metasurface could be used as a highly efficient, ultra-wideband, electrically tunable metamirror in IR and THz frequency bands in several optical devices and systems.

## Methods

In this paper we utilized the Finite-difference time-domain (FDTD) method in order to numerically analyze the anomalous reflection of the electromagnetic waves from the surface of the designed metasurface. Surface conductivity of the graphene sheets for various Fermi-levels in the mid-IR and THz frequency bands are imported to the simulation. Two different simulation approaches were pursued to simulate the plasmonic response of the 20 sheets of the graphene layer realistically; (1) Scaling the conductivity of a single graphene layer to 20, (2) Considering 20 separate sheets of graphene with the 5 nm separation distance between them. The results from both approaches were compared and verified no differences. Both simulation methods confirmed the efficiency and angle of the anomalously reflected light from the metasurface. The complex permittivity function of the gold material for the ground plane reflector is imported from the Johnson and Christy data. The smallest mesh sizes used in the simulation are 100 nm, 80 nm and 2 nm in cubic shape to obtain consistent data. We also verified our simulation results analytically by the numerical method using the equations presented in the context.

## Supplementary information

Supplementary Information
