# Supplementary material for: Ultra-High Efficiency and Broad Band Operation of Infrared Metasurface Anomalous Reflector based on Graphene Plasmonics
Source: Sci Rep. 2019 Feb 4;9:1249. doi: 10.1038/s41598-018-37562-y (PMC6362233; doi:10.1038/s41598-018-37562-y)
Supplement: Supplementary file 1 — Supplementary Information [file 41598_2018_37562_MOESM1_ESM.pdf]

# Ultra-High Efficiency and Broad Band Operation of Infrared Metasurface Anomalous Reflector based on Graphene Plasmonics


**Sina Soleymani[1], M. Zeki Gungordu[1], Patrick kung[1], and Seongsin M. Kim[1,*]**

[1]Electrical and Computer Engineering Department, University of Alabama, Tuscaloosa, 35487, USA

*Correspondence and requests for materials should be addressed to S.M.K.

(email: seongsin@eng.ua.edu).


*Supplementary Information*

**Calculation of Eq. (1) and Eq. (2)**

The Eq. (1) is produced from the circuit model of the graphene ribbons. [37, 38] The resonance frequency could be easily calculated from $\omega_0 = 1/\sqrt{LC}$, where

$$C = \frac{8W^2(\varepsilon_a + \varepsilon_s)\varepsilon_0}{9D\pi P}$$

$$L = \frac{9D}{8W\omega}\frac{1}{Im\{\sigma\}}$$

In which $W$, $D$, and $P$ are the width of the graphene ribbons, periodicity, and the periodicity related factor, respectively. $P = Wq/\pi$, and q is the eigenvalue produced by mode solving of the ribbons. For instance, we can consider the average value of the $P$, equal to 0.65 (i.e $W/D \approx 0.5$), as the proportional value of the $W/D$ is changing over the length of the triangular graphene unit cell. Assuming the range of the $W$ from 2μm to 0.1μm, the wide band of the plasmonic resonance wavelength is supported by the structure. For instance, for the longest plasmonic wavelength supported by the structure could be calculated with $W_{max} = 2\mu m$, where $W_{max}/D \approx 1$, therefore we can consider the proper value in the range of $0 < P < 0.42$.[37] From our simulation results, the proper value of $P$ for computing the longest plasmonic wavelength is calculated to equal $P = 0.225$.

Considering N conductive graphene sheets the conductivity could be scaled as $\sigma' = N\sigma$.[39] Hence, inductance of the multilayer graphene sheets could be calculated from

$$L = \frac{9D}{8W}\frac{\pi\hbar^2}{Ne^2\mu}$$

Accordingly the resonance frequency is given by Eq. (1),

$$\omega_0 = \frac{e}{\hbar}\sqrt{\frac{PN\mu}{W(\varepsilon_a + \varepsilon_s)\varepsilon_0}}$$

The value of the Fermi level is considered to be $\mu = 250mev$. The reason for this accurate choice is to attain the plasmonic response in the wider operating wavelength band. Increasing the value of the $\mu$ provides a better plasmonic response of graphene in Terahertz and mid-IR spectra, by blue shifting the zero-crossing of the imaginary part of the conductivity function. However, this increment also blue-shifts the highest plasmonic resonance wavelength supported by the structure. For instance, considering the structure with $P_x = 20.1\mu m$, $P_y = 2.2\mu m$, and $W_{max} = 2\mu m$, the longest plasmonic resonance frequency (considering the proper value of $P = 0.225$ according to the ratio of W/D) is going to be $\lambda_R \approx 21\mu m$. As it could be easily observed in Figure 4(b), the metasurfaces with the longer cut-off wavelength of the Snell's law (i.e. $P_x = 30.1\mu m$, and $P_x = 40.1\mu m$) have the sharp drop on their efficiency on the wavelengths longer than the longest plasmonic resonance wavelength $\lambda_R \approx 21\mu m$. However, by increasing the $\mu = 500mev$ this resonance frequency drops to $\lambda_R \approx 15\,\mu m$, shrinking the wide supported bandwidth. On the other hand, reducing the Fermi level to $\mu = 200mev$, red-shifts the zero-crossing wavelength of the $Im\{\sigma\}$, which in turn red-shifts the surface plasmon wavelength of the graphene ribbons and prohibits the structure from providing the near-field interaction in shorter wavelengths like $10\mu m$ to $12\mu m$, and red-shifting the shortest supported wavelength ($\lambda_s$), and therefore shrinks the supported wavelength band.

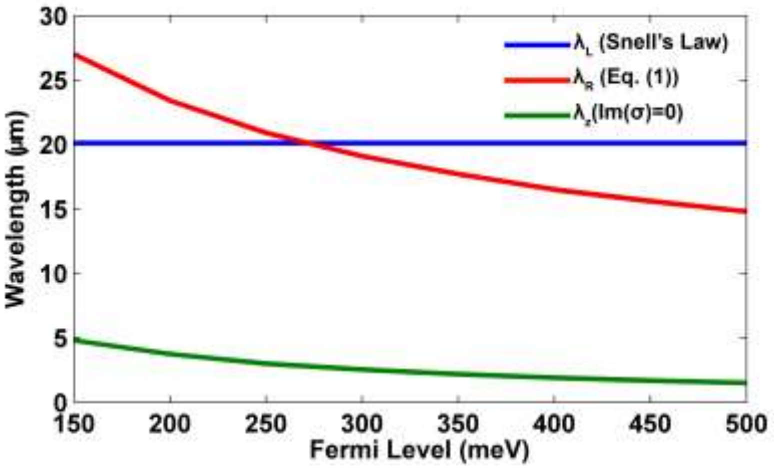


**Figure S 1.** The plasmonic cut-off wavelength ($\boldsymbol{\lambda_R}$) with red color, cut-off wavelength predicted by Snell's law ($\boldsymbol{\lambda_L = 20.1\mu m}$) with blue color, and the zero-crossing wavelength of the imaginary part of the conductivity function of the graphene ($\boldsymbol{\lambda_z}$) with green color.

As it is demonstrated in the supporting figure, the longest resonance wavelength supported by the graphene is reducing by increasing the value of the Fermi level, which shrinks the anomalous reflection bandwidth. In addition by reducing the value of $\mu$ to the values less than 250 meV, the bandwidth does not increase as it is limited by cut-off wavelength of the Snell's law. Moreover, the Cut-off wavelength predicted by Snell's law ($\lambda_L$) is independent from the Fermi level of the graphene and it is related to the length of the Phase gradient unit-cell ($P_x$).

It is worthy to mention that increasing the number of graphene sheets does not change the location of the zero-crossing wavelength of the imaginary part of the conductivity ($Im\{\sigma\} = 0$), and it only intensifies the amplitude of the conductivity function, where it will improve the near-field interaction of the electromagnetic waves.[40]

On the other hand Eq. (2) is presenting the generalized Snell's law.[27]

$$\sin(\theta_r) - \sin(\theta_i) = \frac{\nabla\phi_x}{k_0}$$

Also it is demonstrated that the $k_x = \nabla\phi_x$.[23] Therefore we can write

$$\sin(\theta_r) - \sin(\theta_i) = \frac{\lambda_0}{P_x}$$

**Impact of the Number of Graphene Sheets on the Plasmonic Near-field Interaction and the Efficiency**

Increasing the number of the graphene sheets ($N$), increases the number of the free carriers and conducting channels. This in fact increases the plasmonic resonance intensity and amplifies the plasmonic near-field amplitude.[40, 41] In order to better describe the impact of increasing $N$ in the anomalous reflection efficiency we present additional simulation result. Increasing the number of graphene sheets from 1 to 20, besides providing a phase gradient response over the wide band of the supported plasmonic resonance wavelength (as presented in Figure 2(a)), intensifies the plasmonic response of the material, which in turn increases the efficiency. The efficiency of the metasurface with the unit-cell dimensions of the $P_x = 20.1\mu m$, and $P_y = 2.2\mu m$, and the dissimilar number of the graphene sheets of $N = 20$, $N = 15$, $N = 10$, $N = 5$, and $N = 1$, are obtained with the blue, red, green, black, and brown colors.

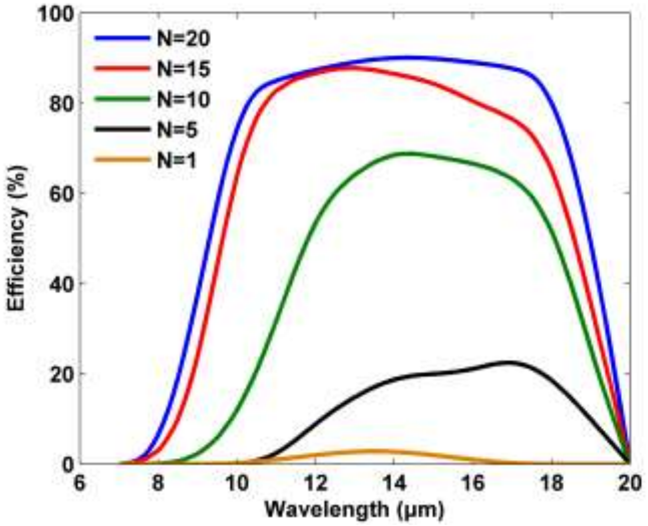


**Figure S 2.** The anomalous reflection efficiency of the metasurface with the unit-cell dimensions of the $\boldsymbol{P_x = 20.1\mu m}$, and $\boldsymbol{P_y = 2.2\mu m}$ , and the number of the graphene sheets of $\boldsymbol{N = 20}$, $\boldsymbol{N = 15}$, $\boldsymbol{N = 10}$, $\boldsymbol{N = 5}$, and $\boldsymbol{N = 1}$, Presented by blue, red, green, black and brown colors, respectively. Incident electromagnetic wave direction is normal to the metasurface $\boldsymbol{\theta_i = 0°}$.

### The Method of the Extraction of the Anomalous Reflection Efficiency

By dividing the value of the reflected power ($R$) to the incident electromagnetic wave's power ($I$) we produce the general reflection percentage, and by considering the percentage of the reflected wave in the anomalous direction with the angle of $\theta_r$ ($R_{\theta_r}$) to the total reflection ($R_T$) we will produce the anomalous reflection efficiency presented in Figure. 4(a), and Figure. 4(b).

$$Efficiency\ (\%) = \frac{R}{I} \times \frac{R_{\theta_r}}{R_T} \times 100$$
